# *Streptococcus pneumoniae* bacteraemia due to parotitis in a patient with systemic sclerosis and secondary Sjögren’s syndrome

**DOI:** 10.1099/jmmcr.0.005068

**Published:** 2016-10-31

**Authors:** Irene Yuen Lin Yii, Jamie Bee Xian Tan, Warren Weng Seng Fong

**Affiliations:** ^1^​ Department of Rheumatology and Immunology, Singapore General Hospital, Singapore; ^2^​ Department of Pathology, Singapore General Hospital, Singapore; ^3^​ Department of Medicine, Yong Loo Lin School of Medicine, National University of Singapore, Singapore; ^4^​ Duke-NUS Medical School, Singapore

**Keywords:** *Streptococcus pneumoniae*, systemic sclerosis, Sjögren’s syndrome, parotitis

## Abstract

**Introduction:**

Invasive pneumococcal disease is an uncommon and notifiable disease in Singapore. It is often associated with significant morbidity and mortality. We report a rare case of invasive pneumococcal bacteraemia due to parotitis in a patient with systemic sclerosis and secondary Sjögren’s syndrome. We also present a retrospective review of *Streptococcus pneumoniae* bacteraemia cases in Singapore General Hospital from January 2011 to April 2016.

**Case presentation:**

A 59-year-old Malay lady with a history of systemic sclerosis with secondary Sjögren’s syndrome presented with fever and left parotid gland swelling. Clinical examination revealed poor salivary pooling and left parotid swelling without fluctuance. Ultrasound of the left parotid gland confirmed acute parotitis without evidence of abscess or sialolithiasis. Blood cultures were positive for *S. pneumoniae*. She was diagnosed to have invasive pneumococcal bacteraemia secondary to acute parotitis, and treated with intravenous benzylpenicillin with clearance of bacteraemia after 3 days. Upon discharge, her antibiotics were changed to intravenous ceftriaxone to facilitate outpatient parenteral antibiotic therapy for another 2 weeks. She responded favourably to antibiotics at follow-up, with no complications from the bacteraemia. A review of the microbiological records of the Singapore General Hospital revealed 116 cases of pneumococcal bacteraemia, most (80.3 %) of which were due to pneumonia. None were due to parotitis.

**Conclusion:**

*S. pneumoniae* parotitis and subsequent bacteraemia is rare. Prompt recognition of the disease and appropriate use of antibiotics are important. This case highlights that close communication between healthcare workers (microbiologist, rheumatologist and infectious disease specialist) is essential in ensuring good clinical outcomes in patients with a potentially fatal disease.

## Introduction

Acute bacterial parotitis is commonly caused by *Staphylococcus aureus* and mixed oral aerobes and/or anaerobes (Brook, 2003). Patients who are debilitated and with poor oral hygiene are particularly susceptible to this condition. *Streptococcus pneumoniae* bacteraemia secondary to *S. pneumoniae* parotitis is rare (Stellbrink *et al.*, 1994; Gomez-Rodrigo *et al.*, 1990; Kataria & Multz, 2009). We describe a patient with a history of systemic sclerosis with secondary Sjögren’s syndrome, who presented with *S. pneumoniae* bacteraemia secondary to acute parotitis.

## Case report

A 59-year-old Malay lady presented with a fever of 40 °C and left parotid swelling of 2 days history. Her medical history was significant for recurrent bilateral parotitis with a background of limited cutaneous systemic sclerosis and Sjögren’s syndrome for 4 years. At the time of presentation, she was not on chronic immunosuppressants. There was no recent history of pneumococcal vaccination.

She had recurrent parotitis that had occurred four times from 2012 to 2014. All episodes required hospitalization for intravenous antibiotics. She previously had *Staphylococcus aureus* and *Klebsiella pneumoniae* isolated from fluid culture from the parotid duct for the recurrent parotitis in 2012. A previous computed tomographyscan of her parotid area performed in 2012 showed left parotitis without evidence of sialolithiasis or abscess.

On examination, there was swelling in the left parotid area without erythema or fluctuance. There were no palpable stones and there was no dental infection or discharge from the parotid duct. Salivary pooling was poor. Systemic examination was otherwise normal.

**Fig. 1.**
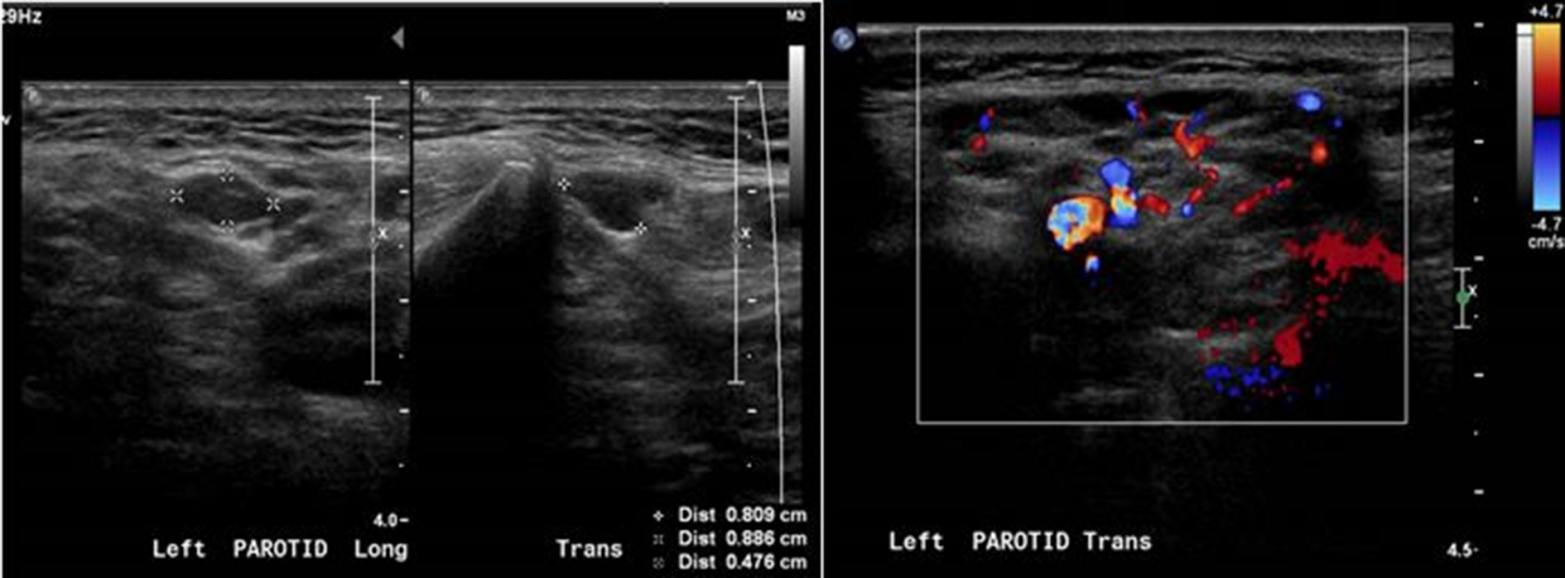
Ultrasound of the left parotid gland showed non-specific inhomogeneous echotexture with multiple small hypoechoic areas and a slight increase in vascularity representing an inflammatory/infective process.

On admission, she had a white blood cell count of 10.78×10^9^ cells l^−1^ (normal range 4.0–11.0×10^9^ cells l^−1^) and a C-reactive protein level of 14.9 mg l^−1^ (normal range 0.2–9.1 mg l^−1^). Ultrasound of the left parotid gland (Fig. 1) showed non-specific inhomogeneous echotexture with multiple small hypoechoic areas and a slight increase in vascularity representing an inflammatory/infective process.

**Fig. 2.**
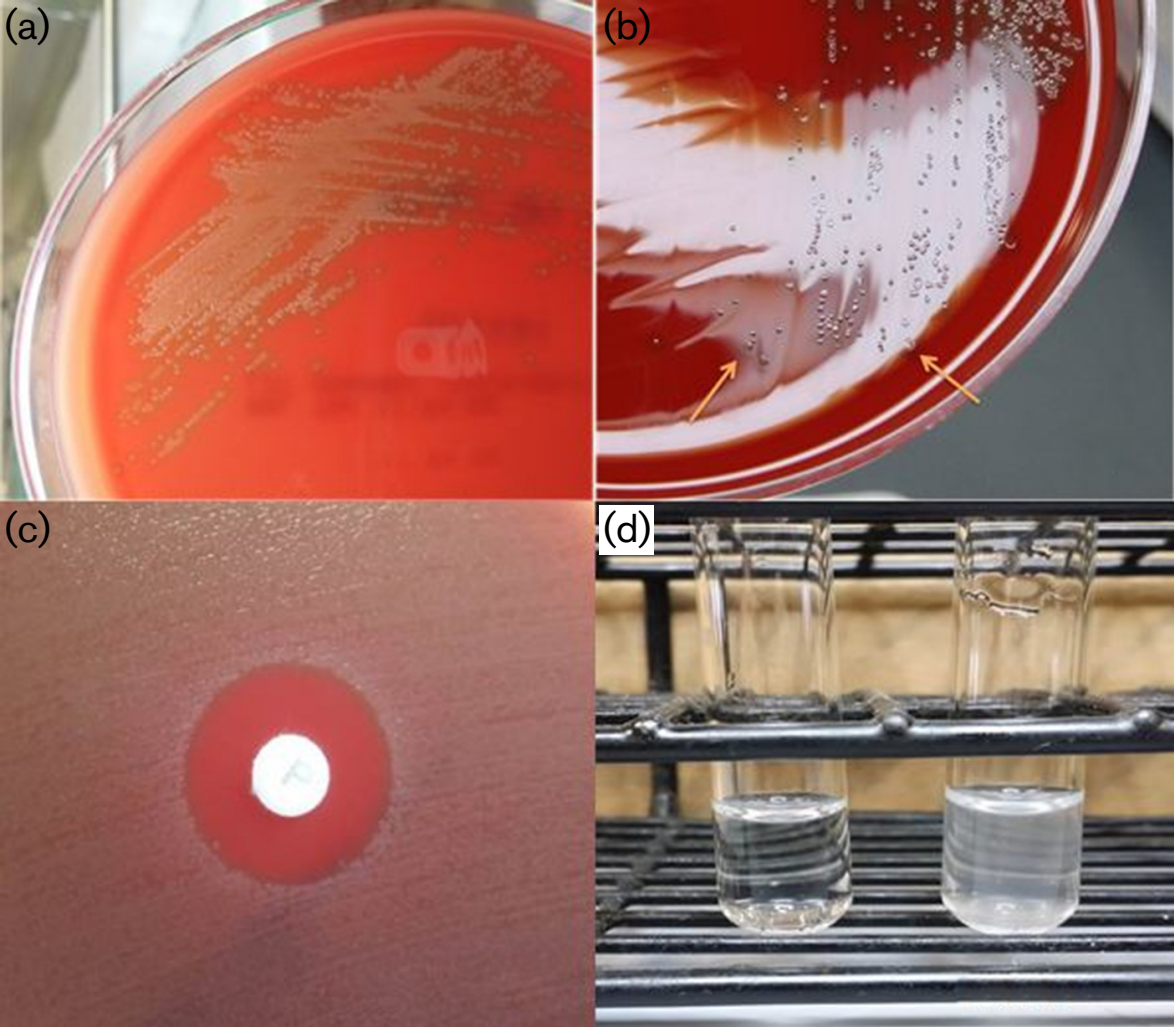
(a) Small α-haemolytic colonies on 5 % sheep blood agar after 24 h incubation. (b) Colonies with a central naval-like depression on 5 % sheep blood agar (see arrows). (c) Colonies were susceptible to optochin. (d) Positive bile solubility test appearing as a clearing of the solution in the presence of bile (left), while the control tube remained turbid (right).

Blood cultures collected on admission were flagged positive after 10 h incubation with Gram stain demonstrating Gram-positive cocci in chains. Small α-haemolytic colonies with a central naval-like depression (Fig. 2a, b) were isolated on 5 % sheep blood agar after 24 h incubation at 37 °C in 5 % CO_2_. These were identified as *S. pneumoniae* based on their being catalase negative, optochin susceptible and bile solubility test positive (Fig. 2c, d). Antimicrobial susceptibility testing was performed and interpreted using Clinical and Laboratory Standards Institute guidelines (CLSI, 2016), which found the *S. pneumoniae* to be sensitive to penicillin (MIC = 0.032 mg l^−1^) and ceftriaxone (MIC = 0.064 mg l^−1^) via Etest, and sensitive to clindamycin, levofloxacin and vancomycin via disc diffusion. In view of the pneumococcal bacteraemia, a transthoracic echocardiogram was carried out and was negative for vegetations. The patient received empirical intravenous ceftriaxone 2g OM initially for 3 days, and this was later changed to intravenous benzylpenicillin 4 megaunits every six hours when blood culture sensitivities were available.

Her fever and parotid swelling improved with antibiotics and subsequent blood cultures were negative. She had received a total of 1 week of antibiotics upon discharge. Outpatient therapy with intravenous ceftriaxone was continued for an additional 2 weeks, with complete resolution of her parotitis. Pneumococcal vaccine was administered to her in the outpatient clinic.

A retrospective review of bacteraemia cases in the Singapore General Hospital from January 2011 to April 2016 revealed 116 cases that were due to *S. pneumoniae*. The majority of these were attributed to pneumonias (94 cases, 81.0 %), followed by meningitis (7 cases, 6.0 %), with isolated episodes attributed to otomastoiditis, retropharyngeal abscess, septic arthritis, spondylodiscitis and spontaneous bacterial peritonitis (5 cases, 4.3 %). None were due to acute bacterial parotitis and no obvious source was found in the rest (10 cases, 8.6 %).

Most cases resolved with treatment and did not develop any complications (93 cases, 80.2 %). Complications such as epidural abscess, spondylodiscitis, and psoas abscess with osteomyelitis and discitis were found in 3 cases (2.6 %), but these patients eventually recovered following surgical intervention and a prolonged period of antibiotic therapy. Twenty patients died, most of these cases were due to pneumonia, whereas only one case was due to meningitis.

A large proportion of the *S. pneumoniae* isolates were sensitive to penicillin, ceftriaxone, clindamycin and vancomycin (71 cases, 61.2 %). Mono-resistance to clindamycin was most commonly encountered (35 cases, 30.2 %). Resistance to β-lactams was infrequently encountered, with intermediate sensitivity to ceftriaxone in 3 cases (2.6 %) and resistance to penicillin in 2 cases (1.7 %). In addition, there were 4 cases (3.4 %) that were intermediately sensitive to ceftriaxone and resistant to clindamycin, as well as 1 case (0.9 %) that was intermediately sensitive to ceftriaxone and resistant to clindamycin and penicillin.

## Discussion

Risk factors for developing parotitis include an immunosuppressed state, dehydration, neoplasm of the oral cavity, recent surgery, parotid duct obstruction and medications like anticholinergic drugs (Brook, 2003). Autoimmune diseases such as Sjögren’s syndrome and sarcoidosis also increase the risk of parotitis (Gomez-Rodrigo *et al.*, 1990). Sjögren’s syndrome is a chronic inflammatory disorder characterized by diminished function of the lacrimal and salivary glands. It is defined as a primary disease or a secondary disease when it is associated with another autoimmune disease, such as systemic sclerosis as in our case. Patients with Sjögren’s syndrome often experience xerostomia and excessive tooth decay (Mathews *et al.*, 2008); hence, these patients should be encouraged to undergo frequent dental consultations and to maintain good oral hygiene.

The most common pathogen isolated from patients with acute parotitis is *Staphylococcus aureus*, but anaerobes are also possible (Brook, 2003). Hence, initial treatment should ideally be broad spectrum to cover empirically for these pathogens. In addition, viruses such as the mumps virus, Epstein–Barr virus, influenza virus, human immunodeficiency virus and cytomegalovirus can also affect the parotid gland (Brook, 2003). *S. pneumoniae*, as depicted in this case, is a rare cause of parotitis (Stellbrink *et al.*, 1994). We did a retrospective review of bacteraemia cases in the Singapore General Hospital from January 2011 to April 2016, which revealed 116 cases that were due to *S. pneumoniae,* but none were due to acute bacterial parotitis. A few case reports have also illustrated the fact that acute bacterial parotitis could initiate the seeding of *S. pneumoniae* into the bloodstream, especially in immunocompromised individuals, mostly in patients with human immunodeficiency virus (Kataria & Multz, 2009; Blanche & Sicard, 1996; Vinasco *et al.*, 2015; Hanekom *et al.*, 1995), but none in patients with Sjögren’s syndrome or systemic sclerosis. Parotitis secondary to *S. pneumoniae* has been reported in a patient with Sjögren’s syndrome (Gomez-Rodrigo *et al.*, 1990), but no bacteraemia was involved.

Neonates and patients of advanced age, and those with chronic systemic disease, immunosuppression and indwelling hardware, are particularly susceptible to developing invasive pneumococcal disease (CDC, 2012; Gentile *et al.*, 2003; Wotton & Goldacre, 2012). Complications include haematogenous spread to the meninges, joints, heart valves and soft tissue. Fortunately, the patient described in our case did not develop complications, such as infective endocarditis, due to prompt treatment with appropriate antibiotics. Studies done on pneumococcal vaccination in healthy adults have shown the vaccine to be 75 % effective in preventing invasive pneumococcal disease (Bonten *et al.*, 2015; CDC, 2010; Kobayashi *et al.*, 2015; Jackson *et al.*, 2003; Moberley *et al.*, 2013). A few studies have been published on the immunogenicity and safety of pneumococcal vaccination in patients with autoimmune conditions (Assen *et al.*, 2011; Elkayam *et al.*, 2002; Mercado *et al.*, 2009; Westra *et al.*, 2015). However, it is unknown how this translates to efficacy in preventing invasive pneumococcal disease in these patients, and more studies will be needed to address this as well as other issues, such as the role of new conjugated pneumococcal vaccines and whether revaccination is required and if so when. Current guidelines from the Centers for Disease Control and Prevention (CDC) have recommended that a dose of PCV13 be followed by a dose of PPSV23 in persons aged more than 2 years who are at high risk for pneumococcal disease because of underlying medical conditions (Kobayashi *et al.*, 2015). Patients with underlying autoimmune disease should be encouraged to receive pneumococcal vaccination in view of the morbidity and mortality associated with *S. pneumoniae* bacteraemia.

### Conclusion

Although it is rare to develop invasive pneumococcal disease from acute parotitis, patients with underlying autoimmune disease such as Sjögren’s syndrome are at increased risk. Blood cultures coupled with imaging studies, followed by the prompt use of antibiotics, should be considered. Close communication between healthcare workers (microbiologist, rheumatologist and infectious disease specialist) is crucial towards ensuring good clinical outcomes in patients with a potentially fatal disease. Vaccination might potentially prevent this disease.
